# Invasive Pneumococcal Disease and COVID-19 With Acute Otitis Media and a Tegmen Tympani Defect

**DOI:** 10.7759/cureus.44869

**Published:** 2023-09-07

**Authors:** Julie Gaudin, Theepha Thayalakulasingam

**Affiliations:** 1 Internal Medicine, Edward Via College of Osteopathic Medicine, Monroe, USA; 2 Pulmonary and Critical Care Medicine, North Oaks Medical Center, Hammond, USA

**Keywords:** covid-19, sars-cov-2, invasive pneumococcal infection, adult bacterial meningitis, pneumococcal bacteremia, tegmen tympani defect, acute otitis media

## Abstract

*Streptococcus pneumoniae *is a leading cause of otitis media, pneumonia, sinusitis, and meningitis. This encapsulated, gram-positive bacterium colonizes the nasopharynx. Major risk factors, including age, hyposplenism, and immunosuppression, predispose to serious infections. Viral infections are known to increase the risk of secondary bacterial infections as the initial immune response can compromise defenses against bacteria. Coronavirus disease 2019 (COVID-19) similarly poses a risk for secondary bacterial infections and coinfections, such as invasive pneumococcal disease (IPD). Still, temporal relationships between IPD and COVID-19 are not fully understood. IPD may also be a complication of untreated acute otitis media. COVID-19 and pneumococcal bacteremia, a form of IPD, have both been shown to damage the blood-brain barrier and gain access to the central nervous system, resulting in deep infections, namely, meningitis and encephalitis. Presented here is the case of a 70-year-old female partially vaccinated against pneumococcal disease, who was initially evaluated for an elevated temperature, acute encephalopathy, and COVID-19. Further investigation confirmed IPD in the form of bacteremia and meningitis. The patient had a protracted disease course complicated by sick sinus syndrome and altered mental status, which led to the identification of otitis media and a right tegmen tympani defect. Emergent implantation of a single-chamber temporary pacemaker and myringotomy with tube placement was performed. Lumbar puncture showed evidence of meningitis. Antibiotic therapy eventually narrowed to ceftriaxone and continued for a total of six weeks. The presence of comorbidities, history of incomplete pneumococcal vaccination series, and concomitant infection with COVID-19 may explain the development of IPD and other complications seen in this case. Furthermore, tegmen tympani defects and damage to the blood-brain barrier can serve as a route for otogenic intracranial sepsis and meningitis. This case serves to reinforce the importance of pneumococcal vaccination and the high clinical suspicion necessary for the prompt diagnosis and treatment of IPD. However, despite vaccination, IPD remains a life-threatening disease due to poor antibiotic penetration in the central nervous system and overlapping presentations with coinfections, such as COVID-19.

## Introduction

COVID-19, caused by severe acute respiratory syndrome coronavirus 2 (SARS-CoV-2), causes systemic manifestations through inflammatory cytokines and binding of angiotensin-converting enzyme 2 (ACE2) receptors in tissues [1]. Similar to other viral respiratory tract infections, evidence suggests that COVID-19 compromises immune defense, increasing the risks of associated infections [2]. In one meta-analysis, secondary bacterial infections were found in 15.5% of COVID-19 patients, while 3.5% reported bacterial coinfections [3]. Associations between COVID-19 and invasive pneumococcal disease (IPD) remain uncommon.

*Streptococcus pneumoniae* colonizes the nasopharynx and commonly causes otitis media and pneumonia. Under immunocompromised states, it can access the bloodstream and enter cerebrospinal fluid (CSF) [4]. Other risk factors for invasive disease include asplenia, CSF leakage from anatomic defects, and damage to the blood-brain barrier due to infections, namely, bacteremia and COVID-19 [5]. IPD is diagnosed with the identification of *S. pneumoniae* in the blood, CSF, or another previously sterile location. The estimated prevalence of IPD is 10.6 per 100,000 persons in the United States [6]. Overlapping clinical presentations, early antibiotic administration, and lack of universal screening may affect prevalence [7]. Furthermore, initial COVID-19 infections may mask symptoms of secondary infections.

Viral respiratory infections also increase susceptibility to acute otitis media (AOM), a middle ear infection [8]. AOM is more common in children, but serious complications are more likely in adults [9]. Although rare, intracranial extension can result in otogenic intracranial sepsis and meningitis, increasing the risks of morbidity and mortality [8]. Abnormalities resulting in communication between the ear and cranial cavity, such as a tegmen tympani defect, serve as a pathway for intracranial extension [5]. Pneumococcal conjugate vaccines (15-valent pneumococcal conjugate vaccine (PCV15) and 20-valent PCV (PCV20)) and pneumococcal polysaccharide vaccine 23 (PPSV23) help prevent complications of pneumococcal disease.

## Case presentation

A 70-year-old female with a past medical history of deep vein thrombosis and obesity and who is a former smoker presented to the emergency department with nausea, dyspnea, and a temperature of 104.1 F. The patient was lethargic and had tested positive for COVID-19 at home one day prior. The patient previously received a complete two-part vaccination series against COVID-19 with an additional booster. The patient received PCV13 alone two years prior to presentation. Physical exam revealed rales and bilateral inflammation of tympanic membranes. Vital signs were significant for tachycardia at 122 beats per minute, tachypnea at 24 breaths per minute, and blood pressure of 124/78 mmHg. Supplemental oxygen was started at 2 L/min nasal cannula to increase oxygen saturation (SpO_2_) from 88% to 94%. Complete blood count (CBC) showed leukocytosis of 12.7 x 10^3^/μL with elevated absolute neutrophil count of 12.0 x 10^3^/μL and microcytic anemia. Laboratory studies showed hypokalemia of 3.3 mmol/L, hyperglycemia of 175 mg/dL, and elevated brain natriuretic peptide (BNP) of 129.5 pg/mL. Lactic acid was within normal limits at 1.29 mmol/L. Chest radiograph (CXR) on admission demonstrated no significant findings. Initial blood cultures suggested gram-positive cocci in pairs and chains on day one of hospitilization. Concern for enterococcal bacteremia led to antibiotic therapy with intravenous (IV) piperacillin-tazobactam.

On day one, the patient was afebrile, and CBC showed worsening leukocytosis of 16.5 x 10^3^/μL with an elevated absolute neutrophil count of 15.5 x 10^3^/μL. The patient was awake, disoriented, and continued moaning. Computed tomography (CT) of the abdomen was performed for concern of an abdominal source of infection and demonstrated no significant findings. Echocardiography demonstrated ejection fraction of 55-65% with no vegetations. Testing for SARS-CoV-2 immunoglobulin G was negative. Further discussion with the patient’s family determined that the signs and symptoms of COVID-19 began two weeks prior to presentation. Isolation precautions were removed following current guidelines. Additional labs showed elevated C-reactive protein at 16.6 mg/L and were positive for rheumatoid factor.

**Figure 1:**
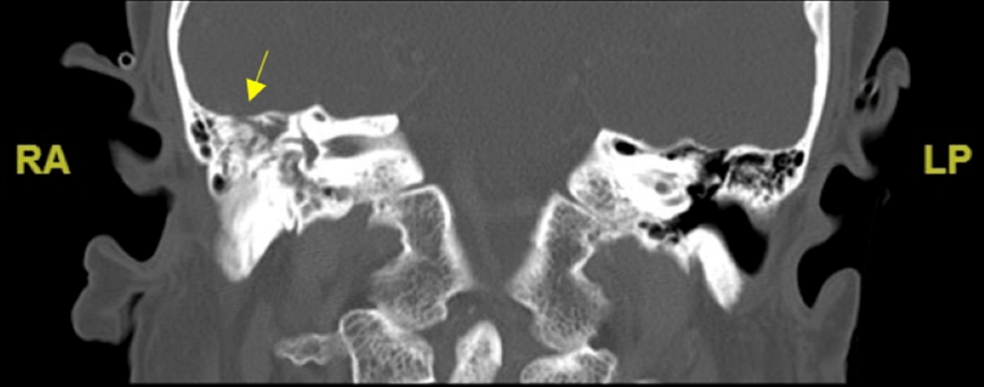
CT evaluation of the internal auditory canal, posterior fossa, and maxillofacial area showing a right tegmen tympani defect and serous effusion, consistent with AOM. AOM: acute otitis media

On day two of hospitilization, vital signs were within normal limits prior to an asymptomatic eight-second pause on cardiac monitor. A second sinus pause lasting 10 seconds led to 75% SpO_2_. The patient became diaphoretic and pale before being stimulated with a response. Emergent placement of a single-chamber temporary pacemaker for sick sinus syndrome was successful. The patient was transferred to the intensive care unit, where she experienced severe headaches and altered mental status. A stat CT evaluation of the internal auditory canal, posterior fossa, and maxillofacial area showed a right tegmen tympani defect and serous effusion, consistent with AOM, as shown in Figure 1. An emergent myringotomy with tube placement was performed, and the patient was started on ofloxacin ear drops. Serous fluid collected from the right ear suggested gram-positive cocci in pairs with abundant leukocytes.

The patient’s mental status (Glasgow Coma Scale score of 11/15) and clinical concern for deep infection led to a medication change to ceftriaxone and ampicillin. A lumbar puncture with fluoroscopic guidance was performed on day four. CSF analysis, as shown in Table 1, showed elevated white blood cell (WBC) count of 2256 mm^3^ with 85% polymorphonuclear leukocytes. CSF cytology showed abundant neutrophils with microscopic examination supporting the diagnosis of bacterial meningitis.

**Table 1:** Cerebrospinal fluid analysis from the lumbar puncture.

Variables	Reference range	Values on presentation
Apperance	Clear	Cloudy, yellow
White blood cell count	<5 mm^3^	2256 mm^3^
Protein	15-45 mg/dL	280 mg/dL
Glucose	40-75 mg/dL	15 mg/dL
Opening pressure	7-18 cm H2O	20 cm H_2_O
Lactate dehydrogenase	<25 units/L	599 units/L

Antibiotic therapy was eventually narrowed to IV ceftriaxone and continued for a total of six weeks. The temporary pacemaker was removed on day nine with resolution of symptomatic bradycardia. The patient had a protracted course, complicated by fevers and continued nausea. By day 12, supplemental oxygen was no longer required, and mentation was at baseline. A peripherally inserted central catheter (PICC) was placed, and the patient was discharged to continue antibiotics in the home setting. Initial blood cultures eventually confirmed *S. pneumoniae.*

## Discussion

COVID-19 is known to cause severe respiratory distress and multi-organ failure through inflammatory cytokines. Increased expression of ACE2 receptors, particularly in the lungs, also provide a route of entry for systemic manifestations [1]. Binding of ACE2 receptors and damage to the blood-brain barrier can lead to nervous system involvement, namely, meningoencephalitis [10]. In one study, this mechanism was suspected to damage the middle ear [11]. Like other viral infections, COVID-19 may compromise immune defense and increase risks of concomitant infections [2].

*S. pneumoniae*, an encapsulated, gram-positive bacterium, colonizes the nasopharynx. In rare circumstances, it accesses the bloodstream and may enter CSF [4]. Meningitis and bacteremia are forms of IPD, which is associated with an increased risk of permanent neurological sequelae and death [6]. Associations between COVID-19 and bacterial infections remain difficult to define due to early antibiotic administration and overlapping clinical presentations. For example, one study found radiological findings between COVID-19 and pneumococcal pneumonia indistinguishable [7]. A delay in diagnosis additionally increases risks of complications from diseases, namely, AOM or pneumonia, that may progress to IPD.

Viral upper respiratory tract infections increase susceptibility to AOM, a non-invasive pneumococcal disease [8]. AOM is often a clinical diagnosis managed in the outpatient setting. Complications occur when AOM is untreated or misdiagnosed, in which infection may extend, causing labyrinthitis or mastoiditis. Much rarer, intracranial extension causes otogenic intracranial sepsis and meningitis [8]. Bacterial meningitis, most often caused by *S. pneumoniae*, is associated with a 30% mortality rate in adults [4]. In one cohort study, *S. pneumoniae* caused 53.8% of 520 adult cases [12]. Headache, neck stiffness, fever, and altered consciousness are common symptoms. Diagnosis requires lumbar puncture with CSF analysis. Risk factors for IPD, including meningitis, are alcoholism, diabetes, asplenia, and immunocompromised status. Damage to the blood-brain barrier and anatomic defects resulting in CSF leakage may provide a route for pathogen entry to the central nervous system. Anatomic defects may be due to congenital abnormalities of the skull, neurotrauma, surgery, or spread of infection from the ears or sinuses [5].

A tegmen tympani defect, as in this case, may provide a route for recurrent otogenic intracranial sepsis. This defect, a tear in the tympanic cavity roof, allows communication between the middle ear and intracranial compartment. On autopsy, tegmen tympani defects are found in 15-35% of patients who presented with otorrhea [8]. Radiologic identification reduces the threshold for the surgical management of AOM. Etiology is often congenital or associated with constant CSF pressure. Idiopathic intracranial hypertension, morbid obesity, or aberrant arachnoid granulation tissue creates constant pressure on the temporal bone from CSF production [8]. Other associations include intracranial herniations, ear surgery, or trauma. In this patient’s case, it is possible that COVID-19 masked the initial symptoms of AOM, leading to the extension of infection. It is unknown if the tegmen tympani defect was present prior to presentation.

SARS-CoV-2 can also enter myocardial tissue and cause direct inflammation and autonomic dysfunction of the sinoatrial node, leading to cardiac manifestations and arrythmias [13]. However, cases of COVID-19 are rarely associated with sick sinus syndrome. Sick sinus syndrome is a disorder of the sinoatrial node resulting in cardiac insufficiency and bradycardia [13]. This condition can be attributed to intrinsic and extrinsic causes, including electrolyte abnormalities, such as potassium.

Current pneumococcal vaccination guidelines from the Centers for Disease Control and Prevention (CDC) include PCV15, PCV20, and PPSV23. If PCV13 was previously received alone, as in this case, PPSV23 is necessary to complete the series [14]. Therefore, the patient had incomplete immune protection against *S. pneumoniae* prior to presentation.

## Conclusions

This case serves to reinforce the importance of pneumococcal vaccination and prompt diagnosis and treatment of IPD. IPD remains a life-threatening disease due to the growing antibiotic resistance, poor antibiotic penetration, and overlapping presentations with coinfections, such as COVID-19. High clinical suspicion of otitis media should remain due to risks of intracranial otogenic sepsis and meningitis.
